# Effect of GGBFS Content and Curing Temperature on Early-Age Strength and Maturity-Based Modeling of Concrete

**DOI:** 10.3390/ma18194525

**Published:** 2025-09-29

**Authors:** Han-Sol Kim, Han-Seung Lee

**Affiliations:** 1 Department of Smart City Engineering, Hanyang University, 1271 Sa 3-dong, Sangnok-gu, Ansan-si 15588, Republic of Korea; abcd0828@hanyang.ac.kr; 2 Department of Architectural Engineering, Hanyang University, 1271 Sa 3-dong, Sangnok-gu, Ansan-si 15588, Republic of Korea

**Keywords:** strength prediction models, sustainable concrete, maturity method, ground granulated blast-furnace slag (GGBFS), early-age compressive strength, datum temperature

## Abstract

This study investigates the early-age compressive strength development of concrete incorporating ground granulated blast-furnace slag (GGBFS) under varying water-to-binder (W/B) ratios (35%, 45%, and 55%) and curing temperatures (5 °C, 20 °C, and 35 °C). Concrete mixtures were prepared with 0%, 20%, and 40% GGBFS replacement levels, maintaining a constant slump of 180 mm. The influence of GGBFS on fresh properties was evident, as higher GGBFS content reduced the demand for high-performance air-entraining water-reducing admixture (AEWR) by up to 72% at 40% GGBFS and W/B of 35%. All mixtures maintained target air content within 4.5 ± 1.5%. The Nurse–Saul maturity method was applied to determine the datum temperature $T_{0}$ (The minimum temperature required for the degree of maturity to increase) for early-age strength prediction. The optimal $T_{0}$ was found to be −3 °C for both OPC and GGBFS-blended concretes, replacing the conventional −10 °C value. Compressive strength predictions were conducted using Plowman, Logistic, and Gompertz models within the 5–10 MPa range. The Plowman and Gompertz models predicted early-age compressive strength with an error of approximately 10% in the 5–10 MPa range. In the lower strength range of 3–5 MPa, the Gompertz model exhibited superior predictive performance, with prediction errors 0.5–1 MPa lower than those obtained using the Plowman model. These findings will help in enhancing the maturity method’s reliability for low-temperature or time-constrained construction and support the use of GGBFS as a sustainable cement replacement. The study offers practical insights into optimizing early-age performance in blended cementitious systems.

## 1. Introduction

The compressive strength of concrete is a key characteristic that influences the structural effectiveness and longevity of building endeavors. This trait is especially important in the early-stage phase, as it directly affects the timing of essential construction tasks like formwork removal. The immediate internal temperature of concrete post-placement greatly influences its compressive strength at an early age, affecting the overall integrity and stability of architectural structures [1]. Precise forecasting of early-age strength enables engineers to refine construction timelines, cut expenses, and improve project efficiency by identifying the exact time for formwork removal while maintaining structural integrity [2]. As a result, considerable effort has been invested in creating dependable approaches for predicting early-age compressive strength, emphasizing non-destructive methods that maintain a balance between precision and practicality [3,4].

A technique known as the maturity method was initially created to evaluate the strength of mass concrete when exposed to cold temperatures. This non-invasive method presumes that the increase in compressive strength is directly related to the combined impact of curing temperature and duration, known as the maturity index. The approach has achieved broad acceptance because of its straightforwardness and relevance to diverse concrete mix designs and environmental factors [5,6,7,8,9,10,11]. The maturity function, initially presented by Saul (Equation (1)), computes the integral of time and temperature during curing, offering a simple measure for strength forecasting. Its incorporation in standards like ASTM C1074 highlights its dependability and user-friendliness in laboratory and field environments [12]. Incorporating the maturity index into predictive models allows engineers to estimate early-age compressive strength more accurately, aiding informed decision-making throughout construction [13].

(1) $$M=\sum_{0}^{t}(T-T_{0})\Delta t$$

In these models, the time-age component at age *t* is represented as a function of temperature-time. In this context, Δ*t* indicates the time unit (h or d), T refers to the mean concrete temperature (°C) within the period, and $T_{0}$ is the baseline temperature (°C). Among the frequently utilized maturity-based models are the Plowman equation, which uses a logarithmic function, and S-shaped curve models like the Logistic and Gompertz functions. The Plowman model, established in ASTM C1074, is commonly preferred for its straightforwardness, needing just the average curing temperature and age of concrete as inputs [12]. This model is especially useful for field applications where intricate measurements are typically unachievable. The Logistic model, initially created to represent population growth, shows a symmetric S-shaped curve that reflects the swift increase in strength development up to half of the maximum strength (S_u_), followed by a slow decline in acceleration. Likewise, the Gompertz model captures processes marked by swift early growth that decelerates with time, making it ideal for modeling the non-linear strength development of concrete [14]. These models accurately depict the usual characteristics of concrete, showing that initial strength increases quickly but levels off as the material develops.

The use of additional cementitious materials (SCMs) like ground granulated blast-furnace slag (GGBFS) and fly ash has grown more common in contemporary concrete mix designs. These materials enhance workability and durability while also tackling environmental issues by lowering carbon emissions linked to cement manufacturing [15,16,17,18]. GGBFS, a by-product from the production of pig iron, is especially appreciated for its capability to partially substitute cement while not greatly affecting mechanical attributes like compressive strength [19,20,21,22]. Moreover, SCMs aid in managing thermal cracking, an essential element in extensive concrete constructions, and boost long-term durability by minimizing permeability and enhancing resistance to chemical assaults [23]. Nonetheless, utilizing SCMs with hidden hydraulic characteristics, like GGBFS, adds challenges in the development of early-age strength. Elevated replacement ratios or reduced curing temperatures can postpone initial chemical reactions, resulting in delayed strength development during the crucial early-age phase [24,25,26,27]. To confront these issues, it is crucial to examine the hydration properties of SCM-blended concretes with different replacement ratios, water-to-binder ratios, and curing temperatures [28,29]. For example, GGBFS shows enhanced reactivity with elevated curing temperatures; however, at temperatures lower than 10 °C, substantial replacement ratios may greatly impede early strength gain, complicating cold-weather concreting efforts. For example, GGBFS shows enhanced reactivity with elevated curing temperatures; however, at temperatures below 10 °C, substantial replacement ratios significantly retard early strength development. Reported results indicate that after 3 days of curing, the strength difference between OPC pastes hardened at 10 °C and 60 °C is approximately 10 MPa, whereas for mixes containing 60% GGBFS, the difference increases to nearly 25 MPa [30,31,32,33]. This pronounced gap highlights the sensitivity of GGBFS systems to temperature during early hydration.

Even with the progress in maturity-based models, a majority of current research concentrates on forecasting compressive strength at times later than the usual 0.5- to 2-day timeframe needed for formwork removal. This emphasis restricts their use in rapid construction schedules, where quick formwork removal is essential for adhering to project timelines. Additionally, the typically accepted datum temperature of –10 °C could be inaccurate within the initial 24 h post-concrete setting, as elevated effective datum temperatures have been noted in certain instances. These differences underscore the necessity for models designed specifically for the early-stage phase, especially for SCM-blended concretes exhibiting varying hydration behaviors. The hydration of cement is a continuous process that proceeds at different rates depending on the cement compounds, water-to-binder ratio (W/B), particle size, temperature, and the presence of admixtures. Generally, higher W/B, larger particle size, and elevated temperatures accelerate the overall hydration rate. Cement hydration can be broadly divided into five stages: an initial heat release due to the reaction of gypsum with aluminate, a dormant period caused by C-S-H film formation, a renewed acceleration of alite hydration leading to the first peak, a subsequent exothermic stage related to C_3_A hydration (second peak), and a final diffusion-controlled stage with decreasing heat evolution [34]. The intensity and timing of these peaks vary with cement type, admixture content, and curing temperature. Blast furnace slag (GGBFS), possessing latent hydraulic properties, does not react immediately with water due to an impermeable surface film, but reacts when this film is disrupted by CaSO_4_ generated during hydration. An increase in the GGBFS replacement ratio results in a relative decrease in OPC content, which in turn reduces the rate of early-age hydration. These characteristics make it difficult to predict the compressive strength (especially the early strength) of SCM-incorporated concrete.

This study aims to investigate the early-age compressive strength (within 2 days) of concrete blended with GGBFS through the maturity approach. Experimental investigations were carried out with different GGBFS replacement ratios and water-to-binder ratios to create maturity–strength curves tailored to this essential phase. These curves were utilized to determine the reference temperature and assess the precision of compressive strength prediction models based on maturity, as detailed in Table 1. This study seeks to improve the accuracy of early-age strength predictions by comparing predicted strengths with measured strengths for the same mix proportions, thereby tackling the shortcomings of current models in fast construction situations. The results will be beneficial to the expanding understanding of SCM-blended concretes, providing useful guidance for enhancing construction methods while encouraging sustainability by lowering cement consumption. It can also serve as a reference for predicting the compressive strength of SCM-blended concretes at early ages.

## 2. Materials and Methods

### 2.1. Materials

The ordinary Portland cement (OPC) used in this study was a commercially available Type I cement, purchased from Sungshin Cement, Seoul, Republic of Korea. This cement is widely used in structural concrete production and conforms to the specifications of ASTM C150 [35]. The ground granulated blast-furnace slag (GGBFS) was obtained from Sungshin Cement South Korea and met the requirements of ASTM C989 [36], which defines the chemical, physical, and performance criteria for slag cement used in concrete.

Both OPC and GGBFS were stored in airtight containers to prevent moisture absorption before use. The chemical compositions of the OPC and GGBFS were determined using X-ray fluorescence (XRF) analysis (XRF, ZSX Primus IV, Rigaku Corporation, Tokyo, Japan). Table 2 presents the oxide composition results, expressed as weight percentages.

**Figure 1:**
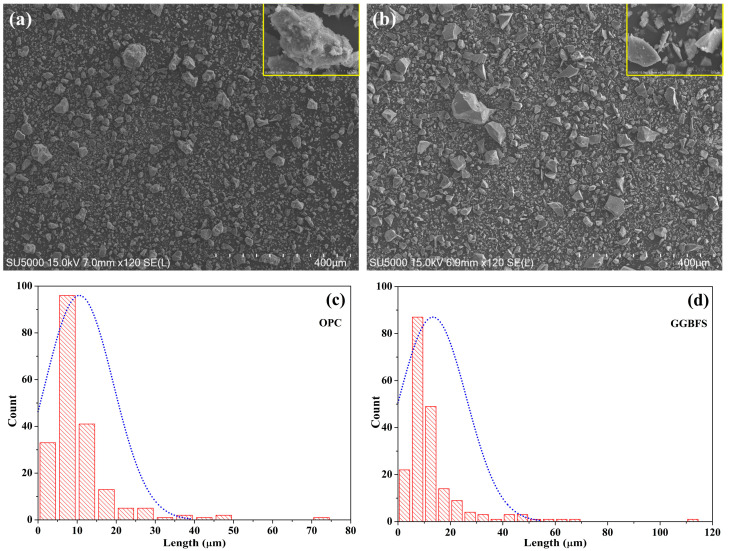
SEM images (**a**,**b**) and particle size distribution (**c**,**d**) of OPC and GGBFS.

The morphology and particle size distribution (PSD) of ordinary Portland cement (OPC) and ground granulated blast-furnace slag (GGBFS) were characterized using scanning electron microscopy (SEM). Representative SEM micrographs of OPC and GGBFS are shown in Figure 1a and Figure 1b, respectively. The micrographs reveal the characteristic angular shape of OPC particles and the relatively smoother surface morphology of GGBFS. Quantitative PSD analysis was performed by processing multiple SEM images using ImageJ software (Version 1.54p). The resulting PSD plots are presented in Figure 1c and Figure 1d for OPC and GGBFS, respectively. The analysis revealed that the average particle sizes were approximately 96 µm for OPC and 87 µm for GGBFS, confirming that the GGBFS used in this study had a slightly finer particle size distribution. This difference in fineness is expected to influence the water demand, workability, and hydration kinetics of the blended concretes, which is consistent with the trends observed in fresh and hardened property measurements.

The OPC had a specific gravity of 3.15 and a Blaine fineness of 3230 cm^2^/g. The GGBFS exhibited a specific gravity of 2.89 and a Blaine fineness of 4330 cm^2^/g, indicating its finer particle size compared to cement, which is advantageous for pozzolanic reactivity.

The fine aggregate used was natural sea sand sourced from [West Sea, Dangjin, Republic of Korea], washed and oven-dried before mixing. Its fineness modulus was 2.80, with a specific gravity of 2.62. The coarse aggregate was crushed granite, sourced from (Sungshin Cement, Republic of Korea), with a maximum particle size of 25 mm, specific gravity of 2.70, and water absorption of 0.8%. The aggregates conformed to ASTM C33 standards [37].

The high-performance air-entraining water-reducing admixture (AEWR) used was a polycarboxylate-based superplasticizer (Flow 3000S, Dongnam, Seoul, Republic of Korea). Clean tap water was used for all mixing and curing processes to eliminate the influence of impurities.

### 2.2. Experimental Variables

The primary experimental variables in this study were the GGBFS replacement ratio and the water-to-binder (W/B) ratio. Table 3 presents the experimental matrix, which comprised three W/B ratios of 35%, 45%, and 55%, three curing temperatures of 5 °C, 20 °C, and 35 °C, and three GGBFS replacement levels of 0%, 20%, and 40% by binder mass. The measurement period for early-age strength evaluation was 48 h. The concrete mix proportions are shown in Table 4, where W represents water, S fine aggregate, G coarse aggregate, GGBFS ground granulated blast-furnace slag, S/a fine aggregate ratio (S/(S + G)) and Ad high-performance air-entraining water-reducing admixture (AEWR). To ensure comparable workability across all mixtures, the AEWR dosage was adjusted according to the mix composition, with particular attention to GGBFS-blended concretes, which generally require less water but may exhibit different flow properties compared to ordinary Portland cement mixtures.

### 2.3. Experimental Method

#### 2.3.1. Methodology for Datum Temperature (T_0_) Estimation

**Figure 2:**
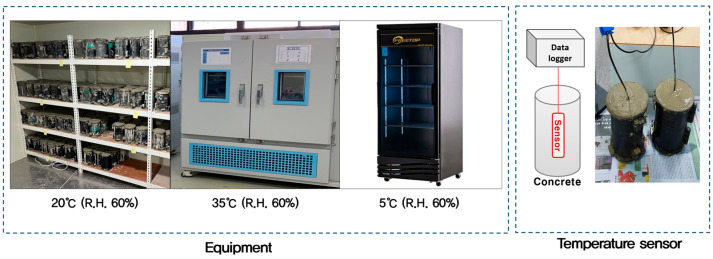
Procedure for determining datum temperature (T_0_) using the Nurse–Saul method.

The datum temperature (*T*_0_) in Equation (1) of the Nurse–Saul maturity method was determined for each mix during the early-age period, up to the point where the compressive strength reached 10 MPa. The testing procedure is illustrated in Figure 2.

Concrete batches were prepared in accordance with Table 4. Immediately after mixing, the fresh concrete was cast into 100 mm × 200 mm cylindrical molds in two layers, each compacted using a vibrating table. The concrete cylinders were capped with gypsum in accordance with ASTM C 617 [38]. Specimens were then placed in temperature-controlled curing chambers set to 5 ± 2 °C, 20 ± 2 °C, and 35 ± 2 °C, with relative humidity maintained at 60 ± 2.5%. These conditions conformed to ASTM C39 [39] curing requirements.

Compressive strength testing was performed hourly after the initial setting using a 30-ton capacity universal testing machine (UTM 30, Woojin, Incheon, Republic of Korea). For each time interval, two specimens were tested, and the average value was recorded.

Age–strength curves were constructed for each curing temperature. Maturity–strength curves were then derived using the Nurse–Saul equation [40].

The datum temperature (T_0_) for each mixture was determined as the value that yielded the highest coefficient of determination (R^2^) between maturity and compressive strength across the three curing temperatures.

#### 2.3.2. Compressive Strength Prediction Models

Using the maturity values calculated from the Nurse–Saul method and the determined datum temperatures, early-age compressive strength predictions were performed with three empirical models—Plowman, Logistic, and Gompertz (Table 1).

**Figure 3:**
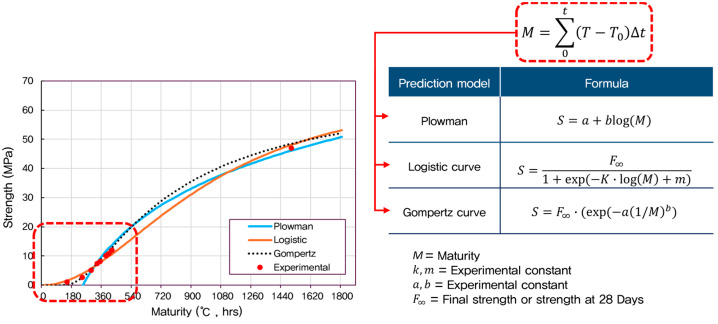
Relationship between concrete compressive strength and maturity for different prediction models.

According to ACI 306R, freezing after reaching 500 psi (3.5 MPa) generally does not affect the strength of concrete. However, if concrete freezes before 500 psi, it risks losing 50% of its original strength. Freshly mixed concrete must be protected against freezing until it reaches 500 psi (3.5 MPa). ACI 301 (Specifications for Structural Concrete) may require higher initial strengths for structures requiring particularly high durability to prevent damage to the concrete surface. In this case, 10 MPa is often used as a safe strength standard to allow removal of lateral formwork (e.g., walls, column sides) [41]. The models were evaluated in the strength range of 4–10 MPa, corresponding to the critical early-age phase important for formwork removal and structural safety. Figure 3 presents a comparison between experimental results and model predictions, showing how each model captures the early-age strength development trend.

## 3. Results and Discussion

### 3.1. Fresh Properties of Concrete

The particle size distribution of cement and GGBFS plays a key role in determining the rheological behavior of fresh concrete. A well-graded binder with a balanced proportion of fine, medium, and coarse particles optimizes packing density, reducing water demand and yield stress while improving flowability. Finer particles contribute to cohesiveness and segregation resistance, whereas coarser particles help maintain fluidity. The relatively uniform PSD of the GGBFS used in this study likely contributed to the observed reduction in admixture demand at constant slump, as shown in Table 4.

The measured fresh properties of all concrete mixtures, incorporating different water-to-binder (W/B) ratios and varying ground granulated blast-furnace slag (GGBFS) replacement levels, are summarized in Table 5. The experimental program was designed to maintain a constant target slump of 180 mm for all mixtures to ensure comparable workability and allow for direct assessment of the influence of binder composition and water content on other fresh properties [42,43].

As evident in Table 5, the required dosage of high-performance air-entraining water-reducing admixture (AEWR) increased as the W/B ratio decreased. This observation is consistent with well-established principles of fresh concrete rheology: a reduction in W/B ratio lowers the volume of free water available for lubrication between particles, thereby increasing internal friction and reducing mobility of the paste [44,45]. Under such conditions, higher doses of water-reducing admixtures are essential to disperse cementitious particles, minimize flocculation, and restore desired workability [46,47].

When comparing mixtures with the same W/B ratio, it is observed that higher GGBFS replacement levels reduced the amount of AEWR needed to achieve the target slump. This effect can be attributed to the intrinsic physical characteristics of GGBFS particles. Unlike clinker particles in OPC, which tend to be irregular and angular, GGBFS particles are typically smoother and more spherical in morphology, with a glassy surface texture [48,49]. These properties reduce interparticle friction and water demand, thus enhancing the fluidity of the mix without the need for excessive chemical admixture dosage [50]. Additionally, the lower specific surface area of GGBFS relative to finely ground OPC can contribute to reduced water absorption at the particle level, further aiding workability [51,52].

The air content of all mixtures fell within the target range of 4.5 ± 1.5%, confirming that adequate air entrainment was achieved across all binder compositions and water contents [53]. Air entrainment plays a critical role in improving freeze-thaw durability, workability, and resistance to segregation and bleeding [54,55]. The consistency in measured air contents across all mixes indicates that neither the reduction in W/B ratio nor the incorporation of GGBFS adversely affected the stability or volume of entrained air under the tested conditions. This stability suggests that the AEWR used in the study was effective in maintaining the required micro-bubble distribution regardless of mix parameters.

These results indicate that, while lowering the W/B ratio necessitates a higher dosage of AEWR to achieve the same slump, the incorporation of GGBFS can offset this requirement due to its favorable particle morphology and reduced water demand. The observed enhancements in fresh concrete properties, particularly the improved flowability with reduced admixture usage in GGBFS mixes, have implications for subsequent strength development [56,57]. In early-age concrete, the initial rheology and mix stability can significantly influence setting behavior and hydration kinetics, especially under variable curing conditions [58]. Therefore, it is important to examine how these fresh state characteristics lead to mechanical performance, particularly compressive strength during the early hydration stages.

### 3.2. Early-Age Compressive Strength Development

According to the guidelines outlined in ASTM C1074 [12], the final setting time of concrete corresponds to a compressive strength of approximately 4 MPa [59]. For the purpose of this study, the onset of meaningful strength development was considered to begin at 5 MPa. The range between 5 MPa and 10 MPa was defined as the critical early-age strength window, as this interval represents the key performance stage related to structural stability and formwork removal in practice. Attaining 10 MPa is often considered the minimum strength required for safe stripping of formwork and supports in field applications [60].

**Figure 4:**
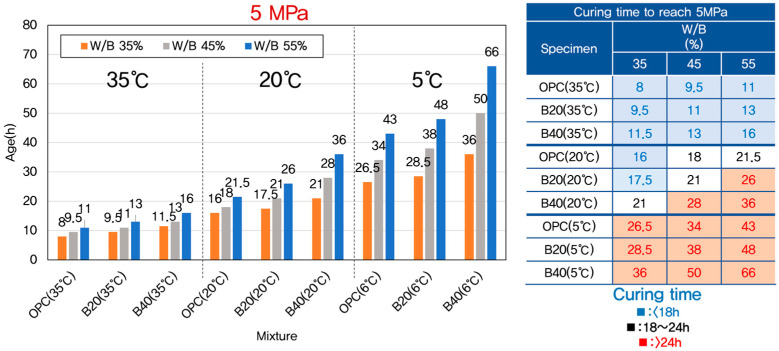
Time required to reach 5 MPa for different W/B ratios, GGBFS levels, and curing temperatures.

**Figure 5:**
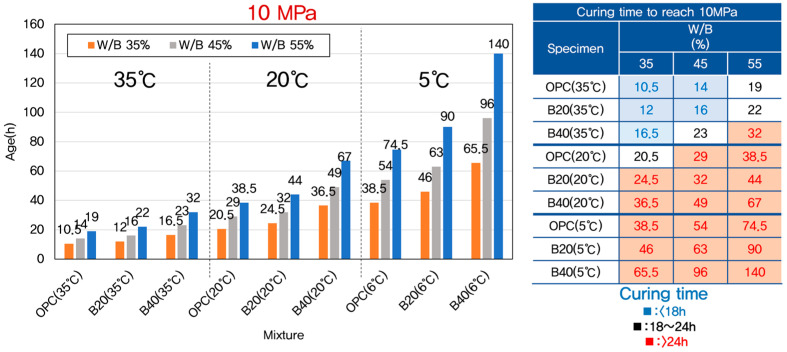
Time required to reach 10 MPa for different W/B ratios, GGBFS levels, and curing temperatures.

The effects of curing temperature and GGBFS (Ground Granulated Blast-Furnace Slag) replacement ratio on the time required to reach these early strength thresholds are presented in Figure 4 and Figure 5. As expected, a strong inverse relationship was observed between curing temperature and the time required to reach both 5 MPa and 10 MPa compressive strength. At elevated curing temperatures (e.g., 35 °C), the hydration of cement proceeded rapidly, thereby accelerating the initial strength gain. In contrast, at lower curing temperatures (e.g., 5 °C), the hydration process slowed significantly, resulting in delayed strength development and extended setting times.

In addition to temperature effects, the replacement of cement with GGBFS had a notable influence on early-age strength development. For a given curing temperature, increasing the GGBFS replacement ratio from 0% to 40% led to a measurable increase in the time needed to reach the strength thresholds of 5 MPa and 10 MPa. This delay in strength gain can be attributed to the intrinsic characteristics of GGBFS. Unlike OPC, which undergoes rapid hydration upon mixing with water, GGBFS exhibits a latent hydraulic behavior. It depends on the presence of calcium hydroxide (Ca(OH)_2_) released during cement hydration to activate its pozzolanic reaction [61,62]. Consequently, the early reactivity of GGBFS-blended concrete is inherently slower, especially during the first 48 h of curing, which is the focus period of this study.

The combination of lower temperature and higher GGBFS content had a synergistic negative effect on early strength development. Therefore, in practical construction scenarios where early-age strength is critical, such as during winter concreting or fast-track construction, it is essential to carefully consider both curing temperature and GGBFS dosage to avoid construction delays.

### 3.3. Determination of Datum Temperature (T_0_)

Following the analysis of early-age strength development, the next critical step involved determining the appropriate datum temperature for use in the Nurse–Saul maturity function [40]. The datum temperature (commonly denoted as T_0_) represents the threshold below which concrete is assumed to gain no strength. It plays a central role in the accuracy of maturity-based strength predictions [63,64].

The datum temperature is the minimum temperature required for the degree of maturity to increase. In Equation (1), if the concrete temperature is lower than the datum temperature, the degree of maturity does not increase. Therefore, the datum temperature can be defined as the temperature at which cement hydration and strength development cease. For example, when the temperature of concrete is 5 degrees Celsius, the maturity increases 3 times faster when the datum temperature is −10 degrees Celsius in Equation (1) than when it is 0 degrees Celsius, which means that the higher the datum temperature, the higher the sensitivity of concrete to temperature, especially to low temperatures. This is consistent with the research results that in low temperature environments, the datum temperature of concrete up to 3 days of curing (−4.9 to 5.1 degrees Celsius) is higher than the datum temperature (−10.3 to 1.9 degrees Celsius) in the test according to ASTM’s Annex A1 [12], which includes long-term curing.

**Figure 6:**
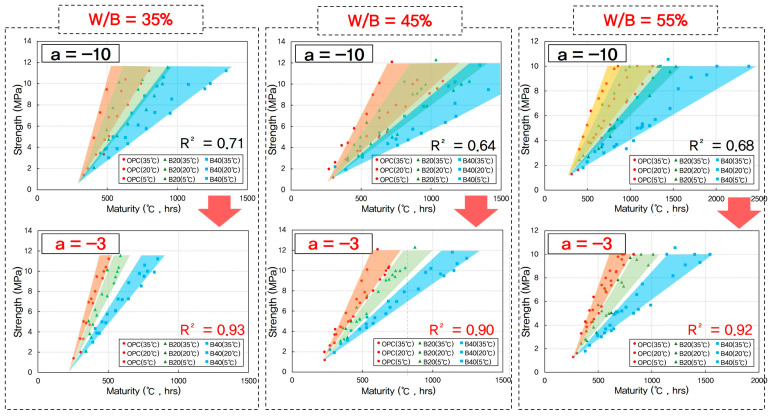
Coefficient of determination (R^2^) for maturity–strength relationships at different assumed datum temperatures.

To identify the optimal datum temperature, compressive strength data for all mixes were plotted against maturity indices calculated using various assumed T_0_ values. Regression analysis was then performed to assess the correlation between maturity and measured compressive strength. As shown in Figure 6, for OPC mixtures, the strongest linear relationship, indicated by the highest coefficient of determination (R^2^), was observed when T_0_ was set to −3 °C. This result is consistent with values reported in the literature for ordinary cement-based concrete, particularly under cold-weather curing conditions [65].

In contrast, for GGBFS-blended mixtures with 20% and 40% replacement levels, the best correlations were obtained at a slightly higher datum temperature of approximately −3 °C. This increase can be explained by the delayed reactivity of GGBFS, which shifts the effective strength development window to higher temperatures compared to OPC [66,67]. However, it is important to note that even for the GGBFS mixes, a datum temperature of −3 °C still yielded a reasonably high R^2^, indicating reliable prediction capability across different binder systems.

Blast furnace slag is a latent hydraulic material that hardens by reacting with calcium hydroxide, which is produced after cement hydrates. Because these secondary reactions are much more sensitive to temperature changes than the hydration reaction of OPC, the datum temperature of concrete using GGBFS is generally higher than that of concrete using only OPC, provided all other mix proportions are the same.

However, experimental results showed that concrete mixed with GGBFS and OPC achieved similar datum temperatures at early ages. This is believed to be due to the use of a higher admixture (water reducer) in OPC concrete to ensure the same workability. Therefore, it was possible to obtain a common value for concrete containing GGBFS mixing of datum temperature.

Therefore, despite minor variation, −3 °C was found to be an effective universal datum temperature for all mixtures studied. The commonly used default value of −10 °C, often applied in standard practice, was found to produce significantly lower R^2^ values and is thus not appropriate for early-age predictions in GGBFS-containing concrete. These findings reinforce the need to calibrate maturity models to specific mix designs and curing conditions to ensure accuracy in field applications.

### 3.4. Prediction of Early-Age Strength Using Maturity Models

**Figure 7:**
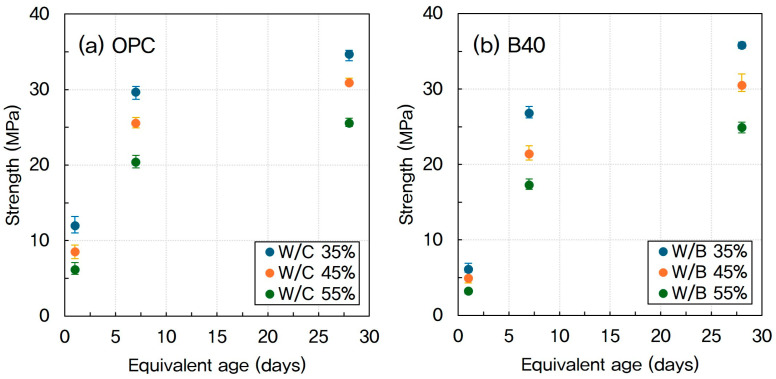
Compressive strength of concrete by GGBFS replacement ratio based on equivalent age (**a**) OPC and (**b**) GGBFS 40%.

Figure 7 shows the compressive strength of concrete at different GGBFS replacement rates based on equivalent age. As the GGBFS replacement rate increases, the development of compressive strength is delayed by up to 7 days.

**Figure 8:**
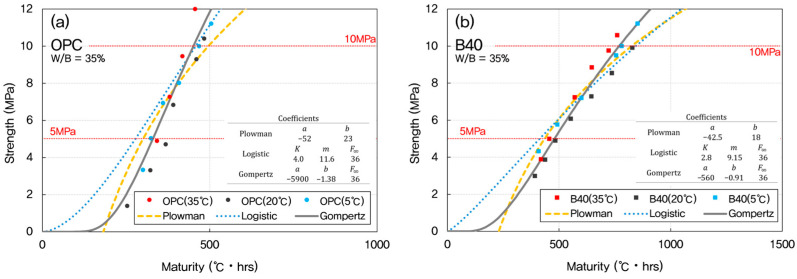
Predicted vs. measured early-age strengths for W/B = 35% using maturity-based models. (**a**) OPC; (**b**) GGBFS 40%.

**Figure 9:**
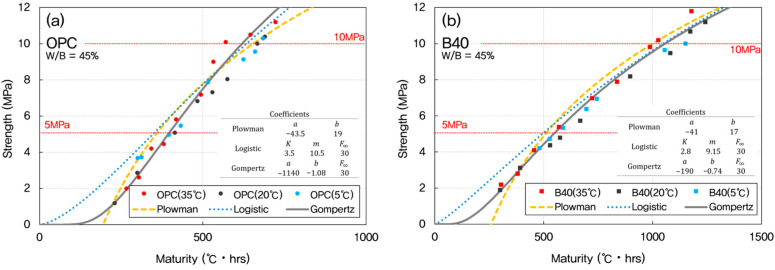
Predicted vs. measured early-age strengths for W/B = 45% using maturity-based models. (**a**) OPC; (**b**) GGBFS 40%.

**Figure 10:**
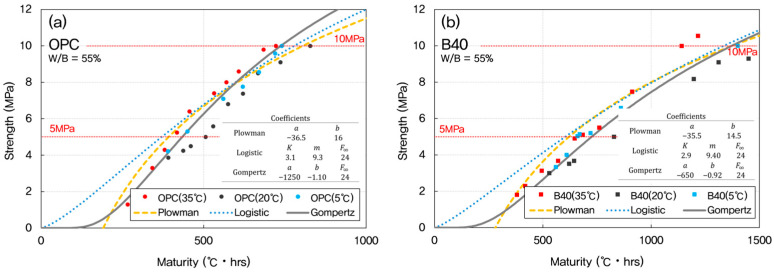
Predicted vs. measured early-age strengths for W/B = 55% using maturity-based models. (**a**) OPC; (**b**) GGBFS 40%.

Once the optimal datum temperature (−3 °C) was established, maturity indices were recalculated and used to predict compressive strength development using three widely recognized models: the Plowman, Logistic, and Gompertz models. These models were chosen for their applicability to early-age strength estimation and their varying mathematical formulations, which allow for performance comparison under different conditions. The predictive performance of each model was evaluated across mixtures with W/B ratios of 35%, 45%, and 55%, as presented in Figure 8, Figure 9 and Figure 10.

For OPC concrete, the Gompertz model consistently provided the closest match between predicted and experimentally measured strengths. Its sigmoidal form effectively captured the nonlinear progression of strength gain characteristic of cement hydration, particularly during the transition from dormant to acceleration phases.

In GGBFS-blended mixtures, both the Plowman and Gompertz models showed good agreement with measured data. The Plowman model, based on logarithmic maturity, is traditionally effective for OPC systems, but its performance in GGBFS mixes was slightly less accurate than the Gompertz model; likely due to the more gradual and extended hydration profile associated with slag. The Logistic model, while still valid, tended to underestimate the rate of strength gain in the 5–10 MPa range, especially in lower W/B systems.

As the W/B ratio increased from 35% to 55%, differences among the three models became less pronounced. At higher W/B ratios, concrete hydration occurs more uniformly, leading to reduced sensitivity in early-age strength prediction. However, for mixes with lower W/B ratios (such as 35%), the sharper strength gradients emphasized the importance of selecting an appropriate model. Here, the Gompertz model showed precision over the others, particularly for accurately capturing early strength behavior in both OPC and GGBFS concretes.

Experimental results showed that early-age concrete exhibited significant variation in setting time and strength, with prediction errors tending to increase with earlier curing ages. The deviation (%) of compressive strength was also greater within 2 days than within 7 and 28 days. This is likely due to the significant variation in initial reaction characteristics depending on the curing temperature and the mixing ratio of chemical admixtures, binders, and other ingredients [68,69].

**Figure 11:**
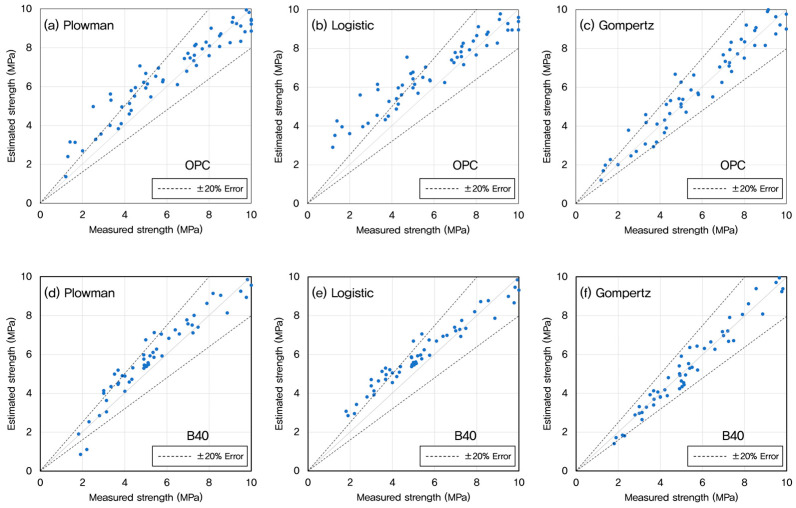
Predicted vs. Measured early-age Strengths for Different Models (**a**–**c**) OPC, (**d**–**f**) GGBFS 40%.

Figure 11 shows a comparison between the measured and predicted strengths depending on the inclusion of blast furnace slag at early age. These results confirm that the Gompertz model is most suitable for early-age strength prediction in a variety of concrete mixtures, particularly when precision is required during the critical 5–10 MPa interval [70]. The Plowman model remains a viable alternative, especially for field applications where simplicity and speed are valued.

While these findings confirm established trends regarding plasticizer demand and temperature-dependent hydration kinetics, this study advances their practical application by quantifying the effects under controlled conditions and integrating them into a calibrated maturity-based prediction framework for GGBFS concretes.

## 4. Conclusions

This study investigated the early-age compressive strength development of concrete incorporating ground granulated blast-furnace slag (GGBFS) under varying water-to-binder (W/B) ratios and curing temperatures. The research focused on identifying a suitable datum temperature using the Nurse–Saul maturity function and evaluating the predictive accuracy of three commonly used maturity-based models: Plowman, Logistic, and Gompertz. Through regression analysis and model comparison, the study aimed to improve early strength estimation, especially for concretes containing GGBFS. The key findings are summarized below:

(i) At a constant target slump of 180 mm, a decrease in W/B ratio resulted in an increased demand for the high-performance air-entraining water-reducing admixture (AEWR). For mixtures with the same W/B ratio, an increase in GGBFS content led to reduced admixture demand due to the smoother texture and lower water demand of GGBFS particles.
(ii) All mixtures satisfied the target air content of 4.5 ± 1.5%, indicating that air entrainment was stable and unaffected by either the W/B ratio or GGBFS replacement level.
(iii) Increasing the GGBFS replacement ratio from 0% to 40% delayed early-age compressive strength development. This delay was more prominent at lower curing temperatures, reflecting the slower pozzolanic reactivity of GGBFS during the initial hydration period.
(iv) Higher curing temperatures accelerated strength development. For example, at 35 °C, both 5 MPa and 10 MPa strength levels were reached significantly faster compared to those at 5 °C, across all mixtures.
(v) The traditionally used datum temperature of −10 °C was found unsuitable for maturity-based strength prediction of GGBFS concretes at early age. A datum temperature of −3 °C produced the highest coefficients of determination across both OPC and GGBFS mixtures.
(vi) Among the models tested, the Gompertz model provided the most accurate prediction of early-age strength. It captured the sigmoidal trend of strength gain more effectively than the Plowman and Logistic models, particularly in low W/B and slag-containing mixtures.
(vii) For GGBFS-blended concrete, both the Plowman and Gompertz models showed reliable predictive performance in the early-age range of 5 to 10 MPa, although the Gompertz model generally offered higher accuracy.
(viii) A single datum temperature of −3 °C is recommended for maturity-based strength prediction of both OPC and GGBFS concretes, making it suitable for practical field applications regardless of binder composition.
(ix) This study supports sustainable concrete practices by enabling reliable early-age strength prediction for GGBFS mixtures and promoting their broader use in structural applications under time or temperature constraints.

Reliable estimates of early-age strength provide the basis for determining the appropriate timing of formwork removal and construction sequence. Determining the optimal formwork removal time can reduce unnecessary delays and overall project duration. This study used Nurse–Saul-based maturity data to predict the early-age strength of OPC concrete and concrete containing GGBFS, with modifications to the datum temperature. However, strength deviations were relatively large in the range below 6 MPa. This suggests that additional early-age testing could be used to expand the data and improve reliability. To verify the applicability of this method to actual construction sites, mock-up experiments simulating field conditions are necessary.

## Figures and Tables

**Table 1:** Concrete compressive strength prediction models using maturity.

Prediction Model	Formula
Plowman curve	S=a+blog(M)
Logistic curve	$S=\frac{F_{\infty }}{1+exp(-K\cdot \log\left(M\right)+m)}$
Gompertz curve	S=F∞·(exp(−a(1M)b)

where S represents the predicted strength (MPa), $F_{\infty }$ denotes the final compressive strength achieved (MPa), a, b, k, and m represent test integers calculated by nonlinear least squares fitting of the actual strength test results by dividing T by maturity and age equivalent.

**Table 2:** Chemical compositions of OPC and GGBFS from XRF analysis (wt%).

Parameter	Phases	OPC	GGBFS
Chemical composition (%)	SiO_2_	21.2	34.0
	Al_2_O_3_	5.03	16.4
	Fe_2_O_3_	3.31	0.50
	CaO	63.18	37.2
	MgO	2.8	6.29
	SO_3_	2.1	2.71
	Na_2_O	0.1	1.33
	K_2_O	0.9	-
Physical properties	Density (g/cm^3^)	3.15	2.89
	Fineness (cm^2^/g)	3450	4330

**Table 3:** Experimental matrix of mix ratios, curing temperatures, and GGBFS levels.

Category	Experimental Level	Unit
W/B	35, 45, 55	%
Temperature	5, 20, 35	°C
GGBFS	0, 20, 40	replacement ratio (% of B)
Measurement period	28	days

**Table 4:** Mix proportions of concrete with varying W/B ratios and GGBFS contents.

No.	Name	GGBFS (%of B)	W/B (%)	W (kg)	S/a (%)	S (kg)	G (kg)	Binder		Ad (kg)
								Cement	GGBFS	
1	OPC-35	0	35	133	51	890	870	380	0	4.3
2	B20-35	20				890	870	304	76	2.0
3	B40-35	40				890	870	228	152	1.9
4	OPC-45	0	45	171	51	890	870	380	0	4.0
5	B20-45	20				890	870	304	76	1.7
6	B40-45	40				890	870	228	152	1.6
7	OPC-55	0	55	181.5	51	890	870	330	0	3.2
8	B20-55	20				890	870	264	66	1.4
9	B40-55	40				890	870	198	132	1.2

**Table 5:** Fresh properties of concrete with varying W/B ratios and GGBFS replacement levels.

No.	Name	GGBFS (% of B)	W/B (%)	Ad (kg/m^3^)	Slump (mm)	Air Content (%)
1	OPC-35	0	35	4.3	180	4.5
2	B20-35	20		2.0		4.0
3	B40-35	40		1.9		3.8
4	OPC-45	0	45	4.0	180	4.4
5	B20-45	20		1.7		4.3
6	B40-45	40		1.6		4.5
7	OPC-55	0	55	3.2	180	4.2
8	B20-55	20		1.4		4.4
9	B40-55	40		1.2		4.3

## Data Availability

The original contributions presented in this study are included in the article. Further inquiries can be directed to the corresponding author.
